# Proceedings of the American Dental Convention. Fourth Annual Session

**Published:** 1858-10

**Authors:** 


### PROCEEDINGS OF SOCIETIES.

### ARTICLE III.

*Proceedings of the American Dental Convention. Fourth Annual Session.*

THE fourth annual meeting of the American Dental Convention, occurred at the Melodeon, in the City of Cincinnati, on Tuesday morning, August 3. The Convention was called to order by the President, Prof. James Taylor, at 10 o'clock, at which time the following gentlemen were in attendance:

NEW HAMPSHIRE.—Dr. Frank Fuller, Portsmouth.

VERMONT.—Dr. M. Tefft, West Poultney.

CONNECTICUT.—Dr. W. Potter, Norwich.

NEW YORK.—Dr. L. W. Rogers, Utica; Dr. W. B. Roberts, New York City; Dr. G. H. Perine, New York

City; Dr. John Allen, New York City; Dr. S. F. Tremaine, Rome; Dr. H. Jameson, Jr., Lyons; Dr. A. Blake, Sardinia.

Pennsylvania.—Dr. Robert Vandervert, Pittsburg; Dr. J. Westbay, Pittsburg; Dr. W. M. Wright, Pittsburg; Dr. S. S. White, Philadelphia; Dr. A. W. Asay, Philadelphia; Dr. W. R. Greenlee, Meadville; Dr. F. J. Chandler, Rochester; Dr. J. B. Williams, Allegheny City; Dr. J. Murray, Beaver; Dr. J. McCalla, Lancaster.

Virginia.—Dr. James Johnston, Staunton.

Missouri.—Dr. Charles Merry, St. Louis; Dr. A. M. Leslie, St. Louis; Dr. Isaiah Forbes, St. Louis; Dr. S. Dunham, St. Louis.

Georgia.—D. S. Chase, Augusta.

Mississippi.—Dr. A. Berry, Raymond.

Tennessee.—Dr. B. Wood, Nashville; Dr. W. H. Morgan, Nashville; Dr. Bryan Cole, Memphis; Dr. C. F. Munster, Jackson.

Kentucky.—Dr. J. B. Lindsay, Maysville; Dr. H. Munro, Lebanon; Dr. W. D. Stone, Lexington; Dr. S. Driggs, Lexington; Dr. D. Dougherty, Bardstown; Dr. James Nourse, Cloverport; Dr. S. Tuttle, Shelbyville; Dr. J. W. Leatherman, Jeffersontown; Dr. Rogers, Shelbyville; Dr. H. McCollum, Augusta; Dr. Geo. P. Newlin, Danville; Dr. R. Peckover, Paris; Dr. B. J. Dudley, Louisville; Dr. J. W. Grant, Lancaster.

Illinois.—Dr. W. W. Allport, Chicago; Dr. I. B. Branch, Galena; Dr. W. P. Kingsley, Freeport.

Michigan.—Dr. P. S. Grimes, Kalamazoo; Dr. John Mansfield, Niles.

Indiana.—Dr. David Cloud, Green Castle; Dr. David Marshall, Carthage; Dr. M. H. Shadoan, Rockport; Dr. J. P. Ulrey, Rising Sun; Dr. W. R. Webster, Richmond; Dr. S. M. Goode, Madison; Dr. J. Hood, Greensburg; Dr. John Keely, Brookville; Dr. George Lupton, Shelbyville.

Ohio.—Dr. C. Palmer, Warren; Dr. A. E. Lyman, Newton Falls; Dr. R. McDowell, Wooster; Dr. S. Brown, Piqua; Dr. Wm. B. Ingersoll, Oberlin; Dr. C. M. Kelsey, Mt. Vernon; Dr. George Watt, Xenia; Dr. S. Clippenger, Bellefontaine; Dr. W. H. Atkinson, Cleveland; Dr. J. Sanford, Chilicothe; Dr. H. A. Smith, Oxford; Dr. J. F. Siddall, Oberlin; Dr. J. Bercher, Findley; Dr. J. L. Dunlap, Chilicothe; Dr. Alfred Terry, Norwalk; Dr. F. R. Rehwinkle, Chilicothe; Dr. Wm. Hall, Piqua; Dr. C. Sill, Franklin; Dr. D. F. Dyche, Lebanon; Dr. Wm. E. Dunn, Delaware; Dr. E. Conway, Dayton; Dr. G. W. Keely, Oxford; Dr. D. W. Harris, West Liberty; Drs. Jas. Taylor, Chas. Bonsall, S. L. Hamlen, B. D. Wheeler, J. Richardson, J. Taft, G. L. Van Emmen, E. W. Ruth, Jas. Erwin, Cincinnati.

The records of the last annual meeting of the Convention were read by the Secretary, Dr. Rogers, of Utica, N. Y., and approved.

The President then presented a letter from the Vice-President, Dr. Elisha Tucker, of Boston, regretting his unavoidable absence and explaining his reasons therefor.

The President remarked that recent severe afflictions had rendered it impossible for him to prepare the customary address, as he had hoped to be able to do. He proceeded, however, in an impromptu speech of much interest, alluding to the history and progress of Dental Education during the past thirty years, and congratulating the profession on the high position to which it had at length attained.

The election of officers was then proceeded with, and resulted in the choice of the following gentlemen:

*President.*—Dr. Isaiah Forbes, of St. Louis.

*Vice-President.*—Dr. L. W. Rogers, of Utica, N. Y.

*Recording Secretary.*—Dr. J. Taft, of Cincinnati.

*Corresponding Secretary.*—Dr. D. S. Chase, of Augusta, Georgia.

*Treasurer.*—Dr. W. B. Roberts, of New York.

The election of officers concluded the doings of the morning session, and the Convention adjourned to meet at 3 o'clock.

---

First day—afternoon session.—The Convention met at 3 o'clock, pursuant to adjournment, and the retiring President appointed Drs. Allport, of Ill., and Chase, of Ga., to conduct the President elect to the chair. Dr. Forbes was introduced to the Convention by Dr. Allport, and on taking the chair, made an appropriate address, thanking the Convention for the high honor conferred upon him, conjuring his brethren to be *honest* in their operations if they would attain to the highest success in their profession, and enjoy that peace of mind which virtuous deeds alone have power to confer. He alluded feelingly to the dispensation of Providence which had recently fallen so heavily upon his worthy predecessor in office, Prof. Taylor, who had just been called upon to follow the wife of his bosom to her final resting place.

The thanks of the Convention were then tendered to the retiring officers, and on motion, it was

*Resolved,* That an Executive Committee of five be appointed by the Chair, to serve during the next year.

The following gentlemen were accordingly selected: Drs. Allport, of Chicago; Wright, of Pittsburg; Leslie, of St. Louis; Fuller, of Portsmouth, N. H., and Stone of Lexington, Ky.

The Executive Committee were requested to examine the accounts of the late Treasurer. After examination, the Committee reported the vouchers correct, and a balance in the treasury of $262 96.

The first topic for debate—"the best means of securing good teeth," was announced as in order by the President. The discussion was opened by Prof. Richardson, of Cincinnati, who read an interesting and instructive paper upon the subject, and was followed by Dr. W. B. Ingersol, in a paper of considerable merit.

Dr. Atkinson believed that the deciduous teeth should be carefully preserved that the permanent may be faultless in position and structure. He advocated filling the primary teeth on the earliest indication of decay.

Dr. Berry thought it important to inquire if in ordinary food we get enough of phosphate of lime to properly develop the bones and teeth. He preferred the "bill of fare" published in Genesis i, 29. He would use unbolted wheat and other cereals.

Dr. Perine believed, from his experience, that much good would result from the use of phosphate of lime exhibited to the mother during pregnancy.

Dr. Rogers did not think it would do to attribute bad teeth to any one cause. In many instances imperfect teeth may be considered hereditary. Improper marriages, the effect of climate, of food, neglect of the organs in early life—all might be cited. He had little confidence in any one theory which proposed to settle this important question.

Dr. Wright thought if he could have the making of all matches for a few centuries, he could ensure a race with good teeth. People would marry, however, "in spite of their teeth." He believed that decay was as frequently internal in its origin as external.

Dr. Watt remarked that though he differed with some who had preceded him, yet he was much gratified with the remarks offered. It was important, he said, to notice two general points. The teeth should be properly developed; and they should be properly protected during development. He did not believe in internal decay of the teeth, and thought it would not do to lose sight of the chemical view of the question. No tooth is so badly developed that it will fall to pieces spontaneously, and none so well that it can successfully resist the action of chemical agents, liable to be brought in contact with it. A well organized, solid tooth, however, would much better resist chemical action, than one otherwise constituted. It was important, then, to pay attention both to mother and child; and this he considered a *practical* question, and practiced accordingly.

The Convention then adjourned to meet at the Lecture Room of the Ohio Dental College, at 8 o'clock on Wednesday.

---

Second day—morning session.—The Convention met at 8 o'clock, agreeable to adjournment.

The first subject, the best means of securing good teeth, being continued, Dr. Frank Fuller remarked, that it was a difficult and interesting question. If we do what we can for the health of the mother, that is all we can accomplish in this direction. He could scarcely understand what was claimed for the phosphate of lime, and would hardly administer it in a state of health, even if the mother's teeth were defective. He recommended a healthy regimen. Bad teeth, he said, were not as usually found among the active, laboring classes, as with those who were confined to bad air, and led artificial modes of life. When the period of eruption arrives, much may be done. The doctrine of internal decay, as suggested yesterday, he considered altogether unsound. The diet he considered was important. The Irish, whose diet is chiefly potatoes, and who lack animal food, have exceedingly brittle teeth, which fracture like glass upon the application of the forceps. He thought this indicated an imperfect condition of the osseous structure—that the earthy and gelatinous matter composing the teeth were not deposited in the best proportions to produce strong teeth. He found these teeth more susceptible to white decay than any other form of caries. An intelligent German in conversation with him remarked that his teeth were good when he came to America, but decayed soon after; and imputed this result to the quality of food used in this country; in the old country he used hard food, which kept the teeth clean; here soft food was provided for him and his teeth decayed. Dr. F. thought the climate had less to do with the decay of teeth than the style of living.

Dr. Atkinson thought that the brittleness noticed by himself and others in the teeth of the Irish, arose from a lack of gelatine and an excess of earthy matter.

Dr. Roberts attributed the prevalent decay in the teeth of Americans, to the mixed races from which we have sprung. This mixture causes a disproportion in the form and size of the teeth and jaws. He did not believe in the theory of internal decay. The enamel, placed upon the outside of the tooth, indicated that the danger is from external causes. Cleanliness was the dentist's *sine qua non.*

Dr. Teft, of Vermont, stated that, through his brother, a foreign missionary, he had some knowledge of the teeth of the Africans, in their native country. In their barbarous state their teeth are good; but, when introduced to civilized life, they decay. He remarked, that on the east side of Lake Champlain, the teeth are attacked with "black decay," while in western New York and Michigan, the white is the prevailing type.

Dr. Rehwinkle said that if the health were perfect, there would be no decay of the teeth. Uncivilized nations live more in accordance with nature. Decay of the teeth was evidently a chemical decomposition. Cleanliness was very important. He thought the use of lime-water and prepared chalk, as prescribed by Dr. White, of Philadelphia, a good suggestion. The constitution of the child might be reached with advantage, sometimes, through that of the mother. He thought an excess of earthy matter rendered the teeth brittle. In Ireland, he said, inflammation of the gums is a prevalent disease. The constitution was often injured by malaria; and the fluids, being thus depraved, act injuriously on the teeth. In the malarious districts of France and Germany, he said, the teeth are not good.

Dr. Kelsey, of Mount Vernon, remarked, that we cannot control marriage,—we must, therefore, take children as we find them. The health of the mother should be carefully watched, and she should have a healthy diet; but, after all, cleanliness is the great thing. He advocated filling the primary teeth, and maintained the chemical theory of decay. Both sets of teeth should be carefully watched, and the primary should not be prematurely extracted. He would not go into the mists of the internal decay question.

Dr. Stone said that the predisposition to decay is oftentimes hereditary, but believed it possible to counteract the tendency. He recommended friction of the gums from early infancy. He referred to the case of a young lady whose teeth were sound at twelve years of age. One year later twelve of them were affected by caries. She drank no water—her diet being chiefly milk. Her saliva was tenacious and ropy. He directed her to abandon the use of milk, and was pleased to find the character of the saliva speedily changed, and the tendency to decay corrected.

Dr. Asay considered cleanliness of the greatest importance. He approved of friction to the gums of infants.

Dr. Branch believed the temporary teeth should be retained as long as possible, and every care taken to secure a healthy constitution in both mother and child.

Dr. Chandler thought the diet had much to do with the condition of the teeth, and had noticed that as a rule in the South, house-servants have bad teeth, and field-hands good ones.

Dr. Taylor said that science had already furnished us with a few leading facts. We know that there are certain elements in the teeth, and that certain articles of diet furnish these elements. Then, different temperaments appropriate and use these elements according to their relative forces. The sanguine temperament, furnished with the same materials, would develop better teeth than the lymphatic. The temperament, however, might change, and the character of the teeth change with it. So far as the dental organs are concerned, he concluded there was nothing deleterious in our climate. The aborigines had good teeth, *even on the salt water coast.* The constitution of the mother influenced that of the child. The blood of a child from a diseased mother, is impaired. An unhealthy mother was not, he said, a suitable or successful nurse. And here a practical question arises. If we cannot impart a normal quality to the mother's milk, we can, at least, recommend that the child be supplied with a healthy nurse. He regarded the phosphate of lime as an important remedial agent, in defects of the osseous tissues. It should be administered, in many cases, where it is deficient in the system. He spoke of its relative quantities in bran and flour, and thought the system would be better nourished by the use of the entire grain. He remarked that the constitution could often be braced up, till the structure of the teeth so improved that they could better resist disease. He remarked that not only the character of the constitution, but even the state of the health could be ascertained by careful inspection of the teeth.

Dr. Rogers, of Cincinnati, regarded this as a most important question, and said that whatever is done, practically, to secure good teeth, must be accomplished in early life. Decay of the teeth resulted from defective enamel, or from vitiated fluids. As the enamel is all-important, our attention should be enlisted during the period of its formation; for it is not changed much after it is formed. The deleterious influence of malaria here, he observed, was similar to its influence in Germany, as described by Dr. Rehwinkle. what can be done to counteract the influence of malaria, he regarded as an important question.

Dr. Murray thought it important to avoid mechanical injuries to the teeth. He had a set of Irish teeth in his head, and he was convinced that with proper care, they would continue to do good service, as they had in the past.

Dr. De Camp thought that physicians should come to our aid, and assist us in making physiologists of mothers, and in modifying and improving public opinion.

Dr. McKellops inquired if decay was chemical solution, why could not chemistry furnish a remedy?

Dr. Dunham believed bad teeth, like diseases, generally, to be hereditary.

Dr. Wright would fill the deciduous teeth when possible, and thus avoid extraction and the consequent contraction of the jaw.

Dr. Allen said we must be careful to avoid extremes. He was in favor of saving the temporary teeth by filling, when it can be done; but there was danger of injury to the permanent organs, by undue pressure. He had met with such results in his own practice.

Dr. Taft said, the remark had been made, that it was proper to retain the deciduous teeth *as long as possible.* When they are not shed at the proper time, making due allowance for constitutional differences, and if the permanent teeth do not follow them directly, he would remove them. They should be retained till the right time; and should not be permitted to become diseased. However, it was often necessary to remove them. When decayed, and he thinks it practicable, he fills them. The pressure *need* be no greater, he thought, than in mastication. Within a year or eighteen months of the period of shedding, he would not consider it proper to fill them; but, three or four years previous to shedding, the attachment is so firm that the pressure need not be dangerous. He considered it a very delicate matter to destroy the deciduous pulp. The parts around a tooth were less likely to remain healthy after the death of the pulp, especially around deciduous teeth. It was important to discriminate. In some constitutions he would not attempt the operation. Indeed, he would only occasionally destroy a deciduous pulp.

The remarks of Dr. Taft concluded the debate upon the first topic, and the second subject for discussion, "The best means of preserving the teeth," was at once taken up.

Dr. Watt remarked that good teeth being "secured," the danger to them arises from the presence of substances, capable of acting chemically upon them. These substances are to be found among the acids. It is true that mechanical injuries and other causes do very much facilitate the action of these agents; yet fracture of the enamel would be a thing of small moment, were none of these agents brought into contact with the tooth. The time was coming, and he believed it was nearly here, when the intelligent dentist would be able to tell precisely what agent produced any given case of decay. When this attainment is made, we can treat the mouth in a rational manner. It was evident, he thought, that many acids, capable of acting on the teeth, have no direct influence in producing dental decay. It was not the brief, accidental presence of acids in the mouth that causes decay, as we ordinarily see it, but the slow and prolonged action of those agents, in a diluted state. Those acids which produce dental decay, he thought, were brought in contact with the teeth by *secretion*, as when the functions of a secreting organ are depraved—by *excretion*, as when the elements which compose them are in excess in the system—by *fermentation* or *putrefaction*, as when particles of food are left to decompose in the mouth. All the four recognized varieties of dental caries can be produced, or at least imitated, artificially, by the use of acids. For example, "white decay" may be readily produced by nitric acid; and as yet he knew of nothing else capable of producing it. If a portion of muscular fibre be left to putrefy, between the teeth, ammonia is invariably formed; but, in the presence of ammonia and atmospheric air, nitric acid is always formed. White decay on approximal surfaces, was thus easily accounted for. Now, constant attention to cleanliness, would remove the particles of food, and also the secreted and excreted acids; but something more, he said, was desirable. It would be well to prevent the formation of the acids. He illustrated this point by allusion to a little dog, chasing away the locomotive. His watchfulness was thus far successful, as it had done no damage; but if the little follow could tear up the track and thus prevent its coming, the course would be more philosophical and satisfactory.

Dr. Berry exhibited a specimen of Japanese tooth powder, which was evidently a pulverised earth—doubtless Armenian bole, or a pulverulent earth, possessing similar characteristics.

Dr. Taft said, that to preserve the teeth, the secretions of the mouth should be kept normal. If they became abnormal, it was important to know how to change them. To remain healthy, a tooth must be exercised. The use of hard, firm food, has a tendency to preserve the teeth. The state of the buccal secretions, have much to do with the question of cleanliness. They were sometimes so adhesive that they attached themselves firmly to the teeth, and were not removed without careful brushing.

Dr. Taylor, in reply to a question of Dr. Stone, entered his protest against the use of candy, but believed that sugar as used in food, had no bad effect upon the teeth.

The discussion of this subject was now closed, and the convention adjourned to meet again at 3 o'clock.

---

Second day—Afternoon session.—The convention met at 3 o'clock, according to adjournment. The third topic for discussion, "the treatment of exposed nerves," was taken up by

Dr. Atkinson, who remarked that, when the nerve was exposed by his manipulations in preparing a cavity for filling, his practice was to apply creosote on a minute pledget of cotton to the exposed portion, making pressure with a blunt instrument for a minute or two: he then capped the nerve with a thin lamina of horn, prepared by beating. He preferred horn to any other substance. This treatment was usually successful, and often resulted in a deposit of cementum by the pulp thus treated.

Dr. Branch had noticed cases in which inflammation had evidently resulted from the use of creosote. If the nerve was lacerated, he waited for the bleeding to subside, and then filled without the use of any escharotic.

Dr. Asay found that inflammation ensued in three-fourths of the cases capped by him. His usual practice was to destroy and remove the pulp, and fill the fang.

Dr. Taft destroyed the nerve with creosote, arsenic, and tannin, and was successful.

Dr. Wright touched the slightly exposed nerves with nitric acid, filled at once without capping, and succeeded in every case, although some discoloration of the tooth occurred from the application of the acid. He had not succeeded well in his attempts to extirpate the pulp.

Dr. Ingersoll reported a case in which he performed what was, at the time, known as "the Hullihen operation." The tooth was a lateral incisor, with exposed nerve. The operation was performed five years ago, and the tooth is still healthy. He had failed in other cases. He had tried capping, but did'nt like it. He now removes the nerve, unless the tooth can be filled without capping.

Dr. Stone said, his practice was to remove as much of the decay as he could, without causing severe pain, and then extirpate. He had quit capping, as he could not get teeth, treated in this way, to last over two years. He removes the nerve with an instrument, wipes out the blood, and fills in a few minutes.

Dr. Perine said, he once extirpated all nerves, but, on receiving suggestions from Dr. Atkinson, he had changed his mode. He thought it important to preserve the vitality of the tooth.

Dr. Johnson, of Indianapolis, said he applied chloride of zinc directly to the exposed nerve, repeating the application if necessary. The pain was severe. Its application, he thought, was more successful with young patients than with old. He had used the chloride in this way for two years, and had seen no discoloration of the teeth.

Dr. Fuller said, he could not get along without the chloride of zinc. It is a good antiseptic, as well as escharotic. He thought it important to fill the nerve cavity. He destroyed nerves with escharotics. He formerly used arsenic and morphine—now he omits the morphine, and finds that less pain is caused by the escharotic action. He now uses chloride of zinc, instead of creosote. In using the latter, the nerve sometimes dies, and the tooth becomes discolored.

Dr. Dunham said, he had capped and failed; and he had succeeded with caps of various materials. On the whole, he preferred plaster of paris as an intervening substance between the nerve and plug.

Dr. Allport could not boast of much success in his attempts to save the vitality of exposed nerves. If at all inflamed, he considered it advisable in all cases to remove it.

Dr. Rogers was convinced, that if a nerve was seriously wounded it would pass on to inflammation and death.

Dr. Rehwinkle thought it difficult to save an exposed nerve. If decomposition of the pulp had commenced, it would be found impossible to restore it to health. If but recently exposed, he would treat with astringents, and fill temporarily.

Dr. Branch would apply gradually increasing cold to benumb the pulp, and then remove it, when indicated. He abandoned the use of morphine, years ago, in his arsenical application, and his patients now complain no more of pain, in destroying the nerve. He succeeds in three-fourths of the cases which he caps.

Dr. Wright wounded the pulp of a molar twenty-four years ago, which bled copiously. He placed a pledget of cotton over the nerve and filled with tin. The operation succeeded, and recently he removed the tin and discovered the cotton in a good state of preservation, and a deposit of cementum over the pulp. The tooth gave every indication of perfect vitality.

Dr. Rogers doubted the diagnosis of Dr. Wright, and was forced to believe that under the circumstances related, pulp must die.

Dr. Berry said, that if he wounded the nerve, he expected it to die. He thought, however, he had seen one successful case in his practice. He extirpates the nerve with an instrument, and said that in the South they had the *nerve* to stand it.

Dr. Palmer said that the pulp may be exposed at minute points, and the operator not be aware of it; and if a little blood be left in the cavity, suppuration, and consequent pressure, would result. In such cases he applied creosote, with a burnisher, over the entire surface. If the exposure be considerable, he would moisten a pledget of cotton with creosote, and press it firmly in the cavity, for some time, then fill up tightly with cotton awhile, and afterwards fill with gold, without a cap. When the nerve is extirpated, he fills the fangs. If the nerve is freely exposed, he applies a muslin cap moistened with creosote, and fills over it. He is opposed to the use of arsenic, though, he said, he was among the first to use it. He thought morphine combined with it, increases the pain. He now uses cobalt, which is milder.

Dr. Roberts said he removes the nerve, when practicable, with an instrument. The operation is not as severe as atients expect it to be. When a front tooth is aching, and he nerve is exposed, he removes it with an instrument, to elieve the tooth-ache, and then fills immediately. The experiment of saving exposed nerves he thought well worth a trial.

Dr. Taylor said his practice was somewhat modified. He was in the habit of capping, more or less. He discriminated more carefully than formerly, but was in favor of capping in appropriate cases. He said it was important to know whether the decay reaches the pulp or not, before we cut down to it. If the tooth is sensitive around the border of the cavity, the nerve, he said, is not exposed. If a nerve was wounded, he formerly destroyed it: but he began to inquire why a wound of the pulp membrane should give more difficulty than cutting off the nerve and vessels in the canal. Reasoning on this, he began to cap and fill, even if the pulp were wounded, and was usually successful in favorable constitutions. In a patient of weak constitution, or badly diseased mouth, he would not attempt this treatment. He used creosote and cobalt in preference to the morphia and arsenic mixture.

Dr. Chase used creosote and arsenic, and obviated pressure upon the nerve by the intervention of a leaden cap. By this means pain was prevented.

Dr. Allen rarely found the application of creosote and arsenic attended with pain, unless the pledget containing it was pressed too firmly into the tooth.

Dr. Osmond found a deposit of osteo-dentine over capped nerves in healthy constitutions. For a cap, he used several thicknesses of gold foil, varnished with gutta percha dissolved in chloroform.

(At this stage of the proceedings, a telegraphic dispatch was received by the President, announcing the death of Dr. C. O. Cone, of Baltimore, when, on motion of Dr. Fuller, it was

"*Resolved,* That as a mark of respect to our deceased brother, the Convention do now adjourn." The Convention therefore adjourned.)

---

Third day—morning session.—The Convention was called to order at 8 o'clock, when the discussion upon the "treatment of exposed nerves" was opened by

Dr. Forbes, who remarked that if the nerve was freshly exposed and uninjured, he would proceed to fill without preliminary treatment. If wounded, he would apply tannin and glycerine. If he cannot save the nerve alive, he preferred to extirpate with an instrument. If his patient was too smart for him, he applied an escharotic.

Dr. Fuller remarked that a nerve could be more completely extirpated when first exposed, than after its destruction; but the removal of a sensitive pulp was found too "heroic" a practice for his patients generally. He was compelled to destroy before removal.

Dr. Kelsey said his patients were "too smart" to have their nerves extirpated with an instrument. He was in the habit of applying chloride of zinc or tannin for the purpose of saving the nerve, but used crude arsenic and chloroform when destruction of the pulp was indicated.

Dr. Rogers, of Utica, suggested that the vitality of the tooth was not lost by the death of the pulp, and that, therefore, these remarks which conveyed that impression, were unguarded or incorrect. In using arsenic to destroy the pulp, he advised coating the surface of the cavity with wax, then displacing a portion of it over the nerve, and applying the escharotic to that point. He thought the tooth was less likely to be discolored. Over the arsenic he applies plaster of paris, when the cavity is accessible, or in such position that he can drop it in. He believed that to fill over the nerve when it is nearly exposed, it will generally die.

Dr. Taft said, that in all that had been said on this topic, we had heard little or nothing in reference to the character of the constitution. As far as described, we would infer that all cases are alike. Now, he said, if we meet with an exposed nerve, in a constitution of low vitality, it is useless to try to save it. But, when the constitution is good, we can save a majority of the cases. In many favorable cases, he said, the nerve would be protected by a deposit of osteodentine. He had frequently found this to be the case on removing fillings to ascertain the condition of the teeth. For caps, he was in the habit of using oil-silk, horn, gutta percha and similar non-conductors. After a tooth was thus filled, however, it might be lost by constitutional disturbance. If the exposure was large, and the pulp healthy, he would generally proceed as above. If inflamed, he would protect it from irritating substances, and apply constitutional treatment; or, if necessary, he would resort both to local and general treatment. The vitality of a tooth, he said, was dependent both on the internal and external membrane. The vitality of the crown was lost by the destruction of the pulp. In reply to a question he said, that when the exposure is slight and the constitution good, nineteen in twenty cases might be saved. He based the statement on actual examination of cases in which he had removed the fillings for purposes of investigation.

The consideration of this subject was now abandoned, and the fourth topic of debate—"Mechanical Dentistry," was announced.

Dr. Rogers, of Utica, called on Dr. Fuller, of New Hampshire, to describe his method of inserting partial sets of teeth on suction plates.

Dr. Fuller responded by stating that he prepares his cast in the usual way, by placing a thin sheet of wax in the plaster arch, of the shape desired for the chamber. After stamping partially, he cuts out the shape of the chamber in the plate, filing the edge square, and strikes up another piece of plate to cover the hole thus formed, thus giving the vacuum a square, sharp border to rest upon the plate. The square edge he considered a great advantage, as the membranes could never be drawn into a chamber thus formed, to any extent. A round edge chamber, on the contrary, soon became filled with the membrane, and there was no longer a vacuum. He never allowed the plate or teeth upon it to impinge upon or touch any of the adjoining teeth.

Dr. Morgan used round edged or swaged chambers in preference.

Dr. Chase did not cut out his chambers, but struck up the border as squarely as possible. Has no difficulty in inserting nearly all his partial sets on suction plates. Did not like the operation of the chamber of Cleveland.

Dr. Murray had acquired an honest prejudice against the "Cleveland" chamber. Had seen a case of fungous growth in a deep chamber of this description. He was able to strike up a chamber with very square borders.

Dr. Allen read a paper upon mechanical dentistry, which contained many valuable hints on the restoration of the form of the face by the "continuous gum" process, which particular idea was illustrated by a most *life-like* and *speaking* figure of a patient before and after being operated upon. It could only be fully appreciated by those who were eye witnesses of the "exposition."

Dr. Roberts described, at length, his manipulations in making continuous gum work, illustrating each part of the process with specimens. He spoke also of the general properties of platinum, and of the difficulty in preparing its scraps for use.

Dr. Vandervert said, that as to the difficulty of uniting platinum scraps, he could weld them together with no other appliances than a blow-pipe, piece of charcoal, a hammer and an anvil. Dr. V. went down to the laboratory of the College with Dr. Roberts, welded two pieces together, and returned. The welded specimen was passed round by Dr. R., and Dr. V. was requested to describe minutely, his manipulation. He said, if he wished to unite a number of scraps, he rolled out the largest one quite thin, and enveloped the others in it, then heated the mass to a high heat, placed it quickly on an anvil and gave it a blow with a heavy hammer, then reheated and gave it another blow, and so on, changing its position at each heating. In a short time the whole mass would be thoroughly integrated, and could be rolled into plate as desired.

On motion, the further consideration of this subject was postponed for the present, and a discussion upon the subject of "filling teeth" was entered upon.

Dr. Atkinson selected for the first operation, the posterior approximal cavity in a left superior second molar. If he could find a reasonable excuse for the removal of the wisdom tooth, he would extract it. If not, he would separate freely and thus obtain tolerably easy access to the cavity. In excavating, he would form grooves or pits as retaining points, insert the gold as usual and finish the plug near the middle, by wedging outward in all directions. If the patient had a profuse growth of hair all around the mouth, he would shield the "imperial" with the napkin, and agglutenize the moustache with accacia mucilage, and draw the mass aside. He used the mallet in condensing, when admissible, and liked it much.

Dr. Allport selected an anterior approximal cavity in the left lateral incisor. He would separate with wedges if possible; if not, he would fill freely; at all events he would have sufficient space. He would insert a bit of soft wood between the necks of the teeth to protect the cavity from the intrusion of the mucous secretions. The great danger of defect was next the gum; and to obviate this, he would insert a large cylinder at this point, and press it firmly into place, with a flat, smooth instrument, leaving a portion of the cylinder projecting from the cavity. Then he would add, successively, soft cylinders, till but a small portion of the cavity, next the cutting edge, is left. This he would fill with crystal gold, or adhesive foil, and but little pressure would be required to render it efficient. He did not say that this would make a *perfectly solid* filling. He did not believe that could be done. He had heard of fillings as solid as molten gold, but he never expected to see any; and fortunately such fillings were not necessary. The tooth could be preserved by one much short of solid gold. He had seen, at this meeting, a fine looking filling, which was perfectly preserving the tooth, which had been inserted by a very distinguished operator, and was yet so soft that it could be pierced by a pointed instrument. He said, that while he was up he would make a remark on the discoloration of crystal gold, and "crystal gold foil." Much had been said in regard to the former changing color; and the charge of impurity had been brought against it. The latter, however, was subject to the same change; and he believed that with both, the fault was not in the material, but in the manipulation. The usual instructions for filling with crystal gold, directed us to begin at the bottom of the cavity, and condense toward the sides, manipulating so as to keep the surface of the filling concave. This, he thought, was incorrect practice. When the surface was concave, he said, that when pressure was made on a piece of gold, in adapting itself to the concavity, it is drawn away from the walls of the cavity. The result is, the filling does not fit tightly to the walls of the cavity, foreign agents are admitted, and discoloration ensues. His mode was to have the filling convex as he progresses,—to keep the plug the fullest in the centre. By this mode, he maintained that the tendency of the gold, under pressure, would be toward the walls of the cavity.

Dr. Stone, in filling a lower bicuspid, would make a bold V shaped separation, leaving a shoulder to prevent the teeth from further approximating. He would fill the lower portion of the cavity with the utmost thoroughness, and finish the plug by wedging from the centre outward.

At 1 o'clock the Convention adjourned.

---

Thursday—afternoon session. The discussion upon the subject of filling teeth was resumed.

Dr. Perine described the process of filling a badly broken incisor, in which he inserted gold wires to aid in the retention of the filling, which was built out with crystal gold.

Dr. Rogers, of Utica, described and recommended an instrument for condensing plugs, after all the gold is introduced. He called it a "serrated burnisher," and said it was made by polishing a serrated or grooved instrument with a brush, and its advantage was, that it condenses the gold without cutting it. The pressure is applied with a kind of "rolling motion." He also suggested the propriety of filling deep cavities partly full of some *non-metallic* substance, before using the gold. It was necessary, he said, to study economy with some patients, and if some indestructible, non-metallic substance could be used, without risk to the tooth, an important point was gained. He had tried two metals in the same tooth, supposing that, if the moisture of the mouth were excluded, no galvanic action would ensue, but he found he was mistaken.

Dr. Stone said, that Dr. Talbert, of Lexington, had experimented in this method, and was successful thus far.

Dr. Dunham thought that many teeth might be saved by filling deep cavities partly full of some soft material.

He reported a case in which osteo-dentine was formed under gutta percha. In small towns especially, some cheap mode is very desirable, if effectual. He thought plaster of paris a convenient and valuable material to place in the bottom of a deep cavity for this purpose.

Dr. Branch said he uses plaster mixed with slaked lime made from marble, for the bottom of the cavity, and fills over it with gold. He uses the mixture to preserve the color of the tooth, and as a non-conductor.

Dr. Tuttle said that in filling the nerve cavity of a front tooth, he would drill through its lingual surface, and fill with the same instruments he would use for a small cavity. He uses serrated points, deep cut, if the instrument be large; not so deep if small.

Dr. Wright said it should be understood that plaster could be rendered no harder by admixture with other substances.

Dr. Berry suggested the use of French plaster, if this substance were used at all.

Dr. Taylor said good fillings can be made in various ways. If he found an operator making good fillings by any one mode of manipulating, he was not anxious to have him change it for another. By request, he described his method of filling a posterior approximal cavity in a lower molar. He would cut away the tooth until he could see the entire cavity. He must have perfect room to work. He often cut away one-third of the grinding surface of the tooth, so that the tongue could displace any food that might lodge in the space. He would form the base of the cavity—the wall next the gum—into an inclined plane, then he would cut under the corners, at the upper portion of the cavity, to form retaining points. He would prepare his gold in blocks or cylinders, using gold sufficiently adhesive to prevent its unrolling. The first block he would have large enough to extend across the entire base, and a little outside of the cavity. Having prepared his blocks of various shapes and sizes, with some formed into cones for finishing with, he would take the first block, and with a flat instrument, press it firmly against the base of the cavity, and, if large enough, it would stay there. Then he would add block after block, selecting shorter ones as he approached the upper portion of the cavity, condensing each block thoroughly as he put it in. If well condensed, they need not project much beyond the cavity. He had nothing peculiar in his mode of finishing. Being requested to make some remarks on filling the fangs of the upper molars, he said he wanted every part of the nerve removed: and his method of obtaining this was, to drill through the centre of the crown, and freely expose the pulp cavity, and then take out the nerve with broaches. He thought young operators often failed by not freely opening the cavity. They were too fearful of cutting away the tooth.

Dr. Lindsay said, he was opposed to wedging the teeth to separate them. There was danger of elongation, or protrusion from the socket, resulting from it. He was in the habit of filing freely; and he never leaves a square shoulder.

On motion, twenty minutes were allotted to the consideration of "local anæsthesia."

Dr. Branch described with considerable minuteness, his method of using his instrument for deadening sensibility, by the action of cold, as a remedial agent; he held that the cold application extended far beyond the extraction of teeth. It was capable of overcoming the sensitiveness of dentine, and inflammation of the gums. He desired to hear from Dr. Taylor concerning it.

Dr. Taylor replied, that his experience had not been uniform. It was at times efficient, but not uniformly.

Dr. Taft found the following mixture, when applied for a minute or two over the region of the tooth to be extracted, an excellent anæsthetic, and used it on cotton.

℞ Tinct. Aconite. Chloroform, Alcohol āā ℥ j.
Morphia Sulphas, gr. vj. Misce.

Dr. Roberts had used electricity in the extraction of about two hundred teeth, with a fair degree of success.

Dr. Dunn said that a lady in Delaware, Ohio, had teeth extracted several years ago under the influence of electricity as directed in the late patent.

The time allotted to this topic having expired, "miscellaneous business" was declared in order.

Dr. Wright exhibited casts of a difficult case of irregularity which he had successfully treated.

Dr. Allport exhibited a set of continuous gum teeth on platinum, which had seriously impaired the patient's health, causing inflammation, and all the symptoms of mercurial ptyalism. Silver and gold plates had been worn with perfect success in the same mouth.

Dr. Rogers reported a case in which similar results followed the use of gold plate. He thought the quality of the plate had nothing to do with the difficulty.

Dr. Watt mentioned a case in which a small twenty carat gold plate produced severe nausea and vomiting—the nausea continuing through a period of some months, as regularly as the plate was worn. The patient was a physician, and was resolved to overcome the difficulty by perseverance. He had not heard for a few weeks, how he was succeeding.

Dr. Dunn exhibited specimens of porcelain work according to Loomis' patent, which he thought had decided advantages over all other styles of work. He maintained that it was cleanlier, stronger, more natural in appearance, easier made, and every way more efficient than other styles.

Dr. Rehwinkle described a case of necrosis of the inferior maxilla, which occurred in his practice. He removed the dead bone, and several teeth connected therewith.

Dr. Conway made some remarks on his dental lamp. This lamp is patented by Dr. C., and is intended to answer all the purposes for which heat is required in the dental laboratory. Dr. C. exhibited its power and uses, at various times, in the laboratory of the college, during the sitting of the convention, and a committee appointed to investigate its claims, subsequently presented a report endorsing its practicability, with some modifications.

Dr. Allport exhibited an appliance, which was successful after other means failed, in retaining a fractured lower maxilla in proper position. In this case the general surgeon recognizing the dental surgeon as his professional brother, applied to him and received his aid.

Dr. Willison reported a case in which a fracture of the jaw occurring in a child, had so changed the position of a concealed first bicuspid, that it made its appearance through the cheek, near the mental foramen.

Dr. Tupton reported a case of filling with amalgam, the cavity being first lined with gold, in which a current of electricity was generated, of sufficient power to cause an unpleasant sensation on touching it with the tongue.

---

Fourth and last day—final session—miscellaneous continued.

Dr. Murray read an interesting paper on diseased antrum.

Dr. Allen described a case of this disease.

Dr. Kilsey had treated three cases of this disease successfully, by injections of warm water followed by a solution of zinc—whether of chloride or otherwise, he did not state.

Dr. Perine reported a case of this disease apparently caused by two large amalgam fillings. The removal of these teeth cured the disease.

Dr. Wright reported a case of diseased antrum in a patient of scrofulous diathesis, which terminated fatally.

Dr. McCalla read a paper on lateral pressure of the teeth.

Dr. McKellops reported a case of protrusion of the lower jaw, which he was correcting by the use of the "Fox bandage," as described in Harris' Principles and Practice.

On motion, one hour was devoted to the subject of filling teeth.

Dr. Palmer described his method of filling an approximal cavity in a front tooth. He separates so as to fill from the lingual surface of the teeth. He discriminates in regard to the different methods of separating. If the necks of the teeth are narrow, he wedges. He makes the cavity triangular, with the apex downward; and in inserting the filling, he finishes at the apex. He polishes the filed surface with pumice, and then with rotten stone.

Dr. Rogers described a file, shaped like a knife blade, which cuts by drawing instead of pushing. With this, he said, it was easy to separate posteriorly, without filing the labial surfaces to any extent.

Dr. Fuller was requested to explain the chief points of difference between the use of adhesive and non-adhesive gold. He accordingly explained that his old method of filling, was, to begin with soft cylinders, prepared according to the dimensions of the cavity, and finish with hard ones, using wedge shaped instruments, throughout. At present he uses Watt's & Co's "crystal gold foil." He makes a groove, or pits, at about the junction of the dentine with the enamel. If the cavity is large he inserts a large pellet, using smaller ones as he progresses. When he gets to the groove, he forces the gold into it, and welds it to the mass which he has built up from the bottom of the cavity. He controls the sublingual ducts by placing a roll of napkin beneath the tongue, and the buccal, by bibulous paper.

Dr. Forbes described his admirable "gouges" for the excavation of cavities.

Dr. Chandler reported a case of hemorrhage of the gums, in which, when every thing else had failed, relief was obtained by a free use of ice-water to the head and neck.

Dr. Rogers considered pressure far more reliable than styptics in ordinary cases.

Dr. Watt said, that the two were easily combined; and in using styptics it was important to make the proper selection. He referred to a case of hemorrhage which occurred in the mouth of a prominent medical teacher. The patient applied pressure and sulphate of copper; and the hemorrhage was arrested only a few minutes at a time. The use of tannin with no more efficient pressure, made, at once, a permanent arrest. The reason of this may be found in the fact, that the salts formed by a union of albumen with the metallic sulphates, nitrates, etc., is soluble in an excess of albumen. Consequently the coagulum would be dissolved, as the flow of blood continued against it. But when the tannic acid is used, the tannate of albumen is formed, which is not soluble in any constituent of the blood.

Dr. Osmond said, it was important, in obedience to the laws of hydraulic pressure, to apply the styptics and pressure directly on the mouth of the bleeding vessel. By observing this, he would not despair of any case.

Dr. Atkinson remarked, that two deaths had occurred in Cleveland, from hemorrhage of the gums during dentition. He spoke favorably of tartaric acid as a styptic.

[This concludes our synopsis of the debates before the convention. The final adjournment occurred at noon, on Friday, Aug. 6, and by a vote of the convention, the fifth annual session will occur at Niagara, on the first Tuesday of August, 1859. In another part of the Journal we have alluded to some matters which came before the convention, but which could not properly find a place in this report.]

